# Supplementary material for: PGRS Domain of Rv0297 of Mycobacterium tuberculosis Functions in A Calcium Dependent Manner
Source: Int J Mol Sci. 2021 Aug 30;22(17):9390. doi: 10.3390/ijms22179390 (PMC8430768; doi:10.3390/ijms22179390)
Supplement: Supplementary file 1 [file ijms-22-09390-s001.zip › ijms-1135486-supplementary.pdf]

Supplementary Information

PGRS domain of Rv0297 of *Mycobacterium tuberculosis* functions in a calcium dependent manner

Tarina Sharma [1,2,†], Jasdeep Singh [3,†], Sonam Grover [3], Manjunath P. [2,3], Firdos Firdos [1], Anwar Alam [2], Nasreen Z. Ehtesham [2,*], and Seyed E. Hasnain [4,5,*]

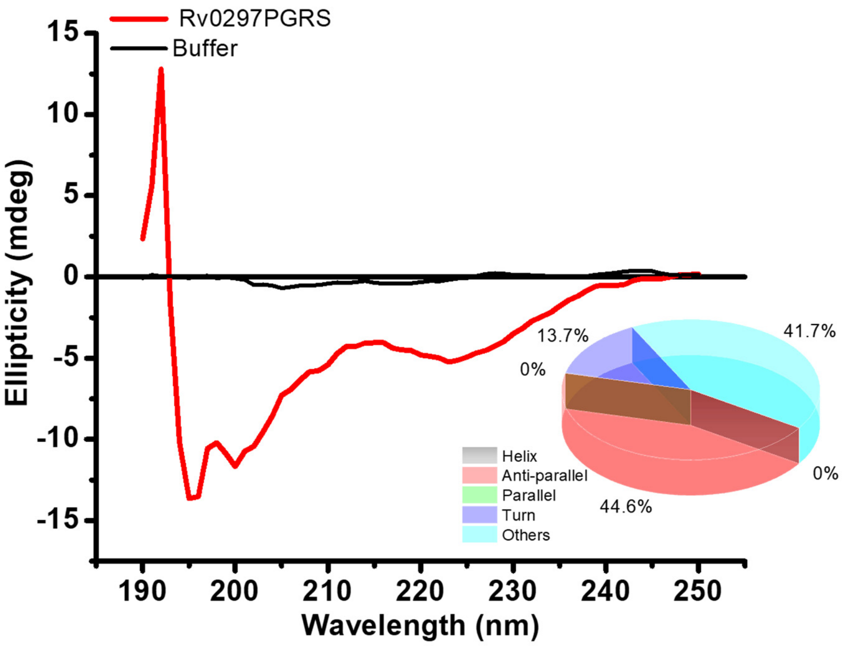

Supplementary Figure S1. Circular dichroism spectra of rRv0297PGRS (Red) in phosphate-buffered saline, pH 7.4. Buffer alone spectra is shown in Black. Inset shows secondary structure content estimated using BeStSel module.

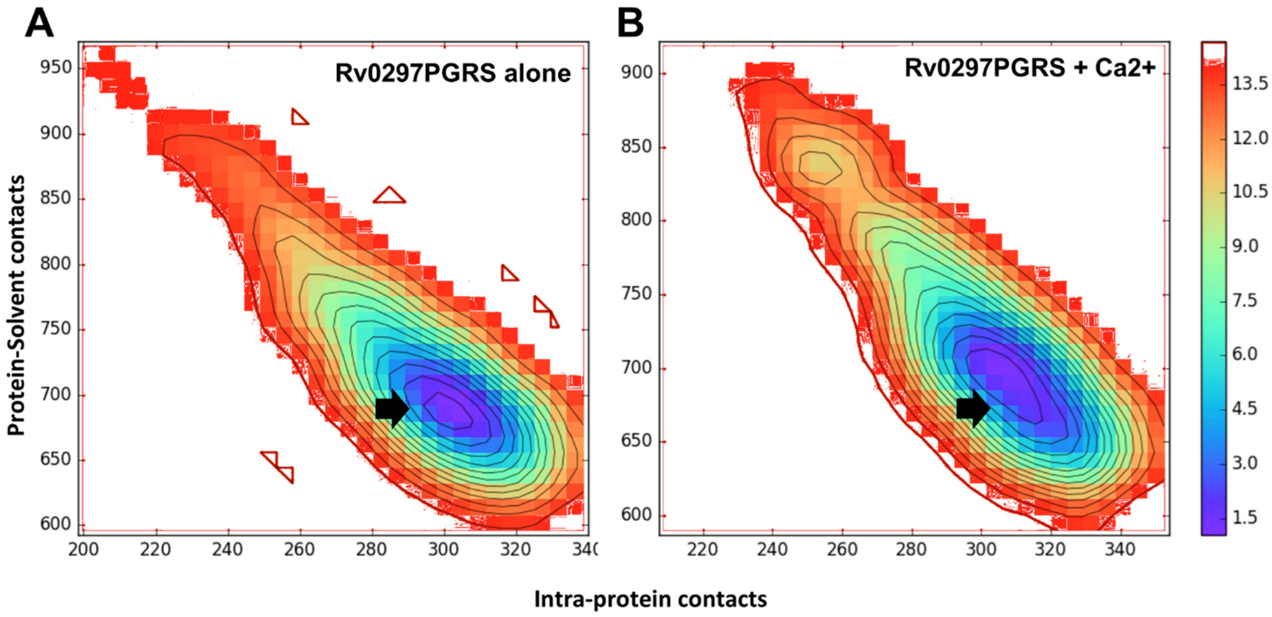

Supplementary Figure S2. Free energy landscapes (in kJ/mol) projected using Intra-protein contacts and protein-solvent contacts. The contacts calculated for (**A**) Rv0297PGRS alone and (**B**) Rv0297PGRS+Ca^{2+} were averaged over the whole simulation trajectory. Black arrows indicate the lowest free energy basin attained by Rv0297PGRS during respective simulations.

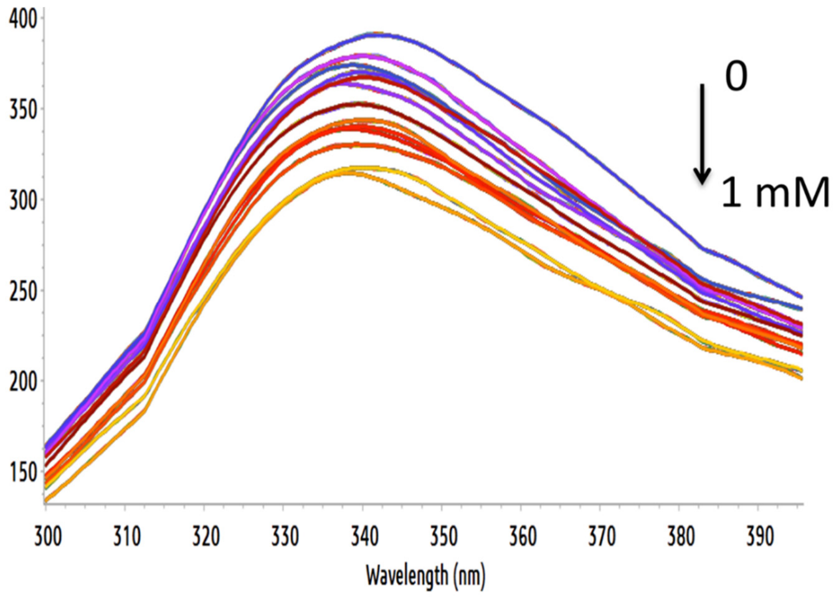

Supplementary Figure S3. Fluorescence titrations of Rv0297PGRS with Magnesium ions. Intrinsic fluorescence emission spectra (300-450 nm) of Rv0297PGRS with increasing concentration of Mg^{2+} in each titration.

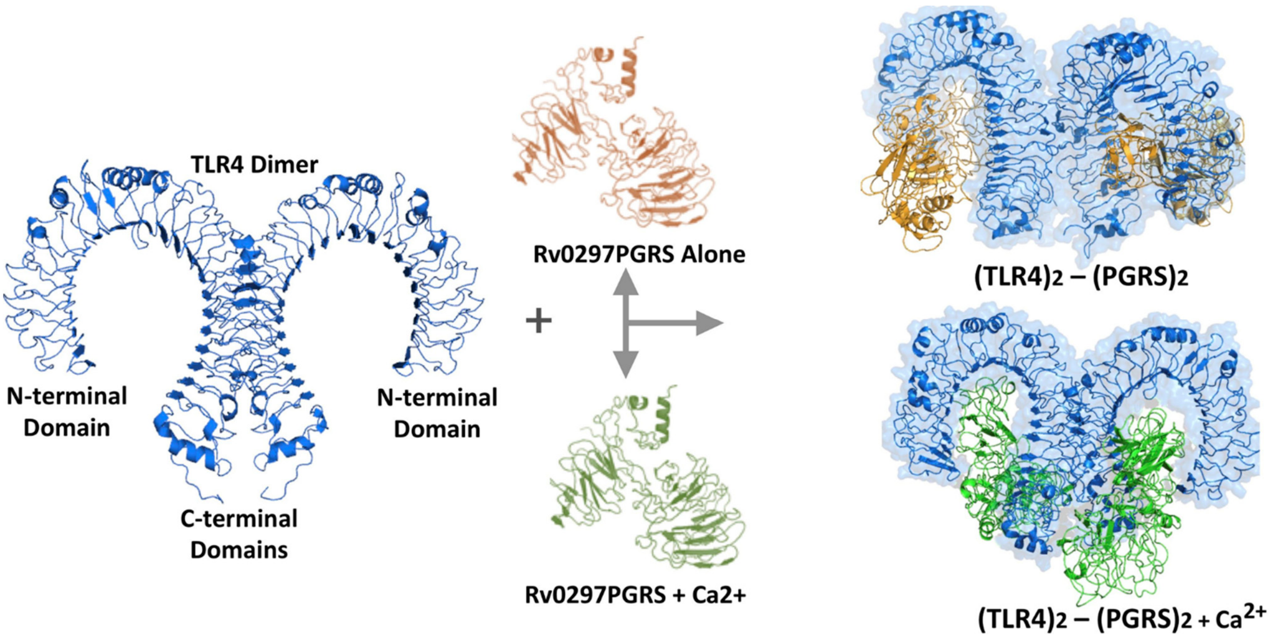

Supplementary Figure S4. Schematic for dimer docking of Rv0297PGRS with TLR4 dimer. Protein-protein docking was carried out using ClusPro and subsequently these models were simulated in the absence and presence of Ca^{2+} ions.

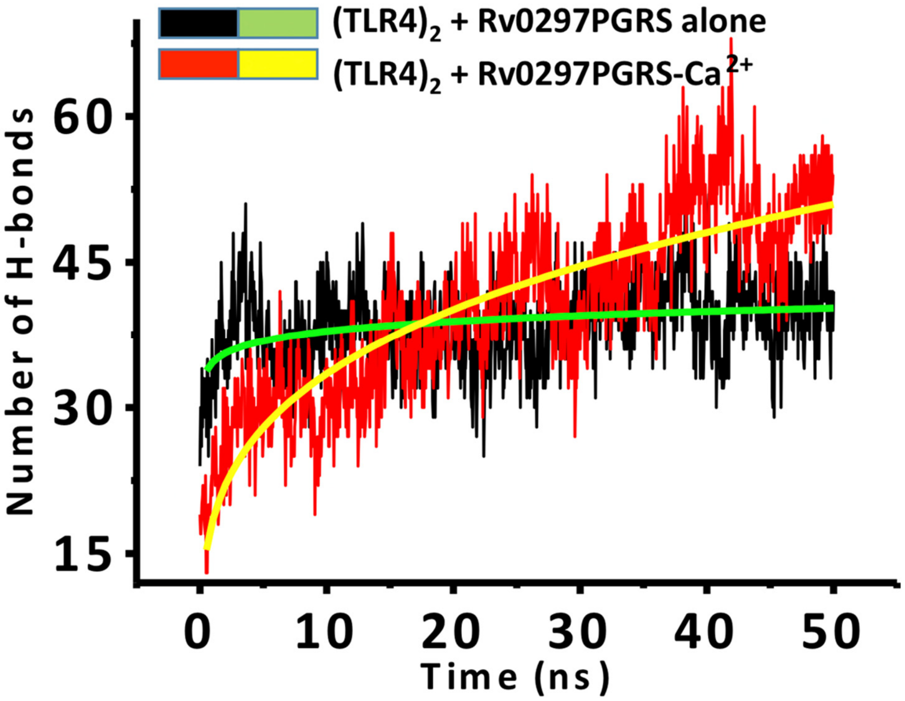

Supplementary Figure S5. H-bond network analysis between Rv0297PGRS and TLR4 dimer. Variations in H-bond number between Rv0297PGRS and TLR4 dimer alone (Black/Green) and in the presence of Ca^{2+} ions (Red/Yellow). Yellow and Green curves are shown to mark differences in the average number of H-bonds per time frame for both systems.

Table S1. List of predicted calcium-binding motifs in the PGRS domains of various PE_PGRS proteins of *Mycobacterium tuberculosis*.

***M.tb* Rv Number**	***M.tb* protein Product**	**No. of putative Calcium binding Motifs**
Rv0109	PE_PGRS_1	4
Rv0124	PE_PGRS_2	7
Rv 0278c	PE_PGRS_3	22
Rv0279c	PE_PGRS_4	15
Rv0297	PE_PGRS_5	15
Rv0532	PE_PGRS_6	7
Rv0578c	PE_PGRS_7	41
Rv0742	PE_PGRS_8	0
Rv0746	PE_PGRS_9	9
Rv0747	PE_PGRS_10	12
Rv0754	PE_PGRS_11	1
Rv0832	PE_PGRS_12	0
Rv0833	PE_PGRS_13	17
Rv0834c	PE_PGRS_14	17
Rv0872c	PE_PGRS_15	9
Rv0977	PE_PGRS_16	18
Rv0978c	PE_PGRS_17	0
Rv0980c	PE_PGRS_18	1
Rv1067c	PE_PGRS_19	16
Rv1068c	PE_PGRS_20	10
Rv1087	PE_PGRS_21	13
Rv1091	PE_PGRS_22	27
Rv1243c	PE_PGRS_23	24
Rv1325c	PE_PGRS_24	9
Rv1396c	PE_PGRS_25	8
Rv1441c	PE_PGRS_26	11
Rv1450c	PE_PGRS_27	44
Rv1452c	PE_PGRS_28	18
Rv1468c	PE_PGRS_29	5
Rv1651c	PE_PGRS_30	12
Rv1768	PE_PGRS_31	13
Rv1803c	PE_PGRS_32	23
Rv1818c	PE_PGRS_33	8

Rv1840c	PE_PGRS_34	10
Rv1983	PE_PGRS_35	1
Rv2162c	PE_PGRS_38	**15**
Rv2340c	PE_PGRS_39	1
Rv2371	PE_PGRS_40	0
Rv2396	PE_PGRS_41	6
Rv2487c	PE_PGRS_42	22
Rv2490c	PE_PGRS_43	74
Rv2591	PE_PGRS_44	9
Rv2615c	PE_PGRS_45	13
Rv2634c	PE_PGRS_46	11
Rv2741	PE_PGRS_47	10
Rv2853	PE_PGRS_48	11
Rv3344c	PE_PGRS_49	23
Rv3345	PE_PGRS_50	74
Rv3367	PE_PGRS_51	18
Rv3388	PE_PGRS_52	18
Rv3507	PE_PGRS_53	67
Rv3508	PE_PGRS_54	56
Rv3511	PE_PGRS_55	25
Rv3512	PE_PGRS_56	54
Rv3514	PE_PGRS_57	38
Rv3590c	PE_PGRS_58	11
Rv3595c	PE_PGRS_59	4
Rv3652	PE_PGRS_60	0
Rv3653	PE_PGRS_61	1
Rv3812	PE_PGRS_62	0
Rv3097c	PE_PGRS_63 ; lip Y	0
